# A comparative analysis of perceptions and evaluations of Simodont® Dental Trainer: a decade of virtual simulation

**DOI:** 10.3389/froh.2025.1646299

**Published:** 2025-08-18

**Authors:** Mahmoud M. Bakr, Andrew Cameron, Ghassan Idris, Mohamed Shamel, Mahmoud Al Ankily

**Affiliations:** ^1^ School of Medicine and Dentistry, Griffith University, Gold Coast, QLD, Australia; ^2^ Children’s Oral Health Service and Child Specialist Services, Metro North Hospital, Queensland Children’s Hospital, Brisbane, QLD, Australia; ^3^ Oral Biology Department, Faculty of Dentistry, The British University in Egypt, El Shorouk, Egypt

**Keywords:** Simodont®, virtual simulation, dental education, comparative analysis, questionnaire, survery, perception

## Abstract

**Introduction/background:**

Virtual dental simulators with a haptics component have been used with great success in dental education for over a decade and is becoming an integral part of dental curricula. A large number of studies have been published about the users’ perception, acceptance and attitude towards virtual dental simulators. However, no longitudinal or long-term studies to our knowledge have been conducted to evaluate the users’ acceptance over time.

**Aims and objectives:**

The aim of the present study is to compare the students’ and academic staff's perceptions and expectations before using the Simodont® Dental Trainer and their evaluation of the technology after using it now vs. ten years ago.

**Materials and methods:**

The participants were invited for a trial session on the Simodont® Dental Trainer and were asked to evaluate different aspects of this virtual simulator by completing a pre-experimental and post-experimental questionnaire. The data collected from the current study was compared against similar data collected and published a decade ago in the same educational institution.

**Results:**

In general, participants from the present study rated different aspects of Simodont® dental trainer higher than their counterparts from the previous study a decade ago. Students from the present study were more excited and more likely to accept the technology when compared to the academic staff members as well as their counterparts from the 2014 student cohort. Academic staff and students from both studies were in total agreement that the Simodont® dental trainer should not be replacing traditional teaching. Both cohorts agreed that the feedback provided by the simulator should be supplemented by feedback from human supervisors. The open-ended responses highlighted the cost-effectiveness, student engagement, value of accessibility and flexibility in virtual dental training as well as persistent technical issues within the software as well as the need to develop more didactic content alongside the practical tasks offered by the simulator.

**Conclusion:**

Generational differences as well as technology limitations affect the perception to virtual dental simulation. The acceptance of the Simodont® dental trainer is following the cycle of technology adoption models.

## Introduction

In an era of rapidly advancing educational technology, virtual simulation has emerged as a transformative tool in dental education (1). Traditional methods, while foundational, often fall short in providing students with the depth, repetition, and realism needed to develop clinical competence. Virtual simulations offer immersive, interactive environments that allow learners to refine their skills without the ethical or logistical limitations of working with real patients. This shift is not merely technological, but also it represents a pedagogical evolution that aligns with modern educational demands (2).

Growing evidence has been compiling for over a decade to support the role that virtual dental simulation plays in dental education (1, 3). The applications of virtual dental simulation are growing from preclinical early psychomotor skills development to include applications through different stages of the dental curricula as well as different disciplines/specialities of dentistry (2, 4, 5). and postgraduate programmes (6, 7). The increased popularity and the diverse application of virtual reality in dental education paved the way for a global VR-Haptic Thinkers Consortium to be established last year (3).

Traditional manikin-based dental simulation has long been a staple in dental education, offering tactile feedback and allowing students to practice hand skills in a controlled, physical environment. However, it lacks the variability, real-time feedback, and adaptability found in more advanced systems (1, 2, 5). The Simodont® Dental Trainer represents a significant advancement, providing high-fidelity virtual simulations with detailed 3D imagery and objective performance assessments (1). Yet, it may still be limited by hardware accessibility and cost (2, 4). Virtual simulation systems, particularly platforms like the Simodont® Dental Trainer, offer a highly immersive experience by replicating real clinical scenarios with visual, auditory, and sometimes haptic feedback, enabling students to engage more deeply with the learning process and refine their skills in a dynamic, responsive environment (1, 5).

A number of studies have assessed the validity of the Simodont® Dental Trainer for over a decade and agreed in concept that it is a valuable tool to be added to the existing traditional methods of preclinical dental education (8–11). Comparative studies in the field of virtual dental simulation have compared between the efficacy of virtual simulation vs. traditional methods of preclinical teaching (12, 13). However, to our knowledge, there are no studies in the literature that investigated the difference between users' acceptance and perceptions of virtual dental simulation over a linear period of time. The aim of our study is to investigate whether there is a difference between students' and academic staff's perceptions of the Simodont® Dental Trainer at present vs. a decade ago.

## Materials and methods

The study was approved by the Griffith University Human Ethics Committee (GU Reference Number: 2025/146). The present study protocol was designed to mimic previous published studies conducted within the same educational facility (8, 9).

All first-year students in the first semester of study to ensure that they are handpiece naive and academic staff members within the School of Medicine and Dentistry – Griffith University were invited to participate in the study. The selection criteria for participation were consistent with our studies that were conducted and published a decade ago (8, 9). Forty first year students and eleven academic staff members with no previous experience in virtual dental simulation from the School of Medicine and Dentistry (Griffith University) were recruited to participate in this study on a first come first served basis to keep the sample size consistent with the previous studies conducted in the same educational facility over a decade ago (8, 9), to evaluate the fidelity of different aspects of the Simodont® Dental Trainer and to assess its value as a new tool in preclinical dental training and early development of Psychomotor skills.

All participants were asked to complete a pre-experimental questionnaire (Table 1) that was designed by educational advisors from ACTA (Academic Centre for Dentistry in Amsterdam) and validated in previously published studies (8–10). The pre-experimental questionnaire consisted of a series of seven questions on a 5-point Likert scale detailing their impression and expectations towards the Si­modont® Dental Trainer. Furthermore, all participants were asked to complete a post-experimental questionnaire (Table 2) that consisted of sixteen questions on a 5-point Likert relating to their experience with the Simodont® Dental Trainer and their opinions about its value in preclinical dental training and the development of psychomotor skills for early learners. The post-experimental questionnaire was also validated in previously published studies (8–10). Following completion of the post-experimental questionnaire, participants were invited to comment about the advantages, limitations and missing elements in the Simodont® Dental Trainer through open-ended questions. The responses from the pre-experimental questionnaire were compared the responses to the first eight questions of the post-experimental that explored the same concepts using a paired sample t-test. Furthermore, the responses from the current pre-and post-experimental questionnaires were compared to the responses from the corresponding questionnaires from the previous studies conducted within the same educational facility involving academic staff (8) and students (9)using a paired sample t-test. Finally, the responses to the open-ended questions were analysed using manual thematic coding in the form of open, axial and selective coding to identify and quantify themes and repeated patterns within the responses as well as link them to user groups (students vs. academic staff).

**Table 1:** Showing means and standard deviations, and frequency distributions of answers of the students’ pre-experimental questionnaire.

Question	Mean	SD	Strongly disagree (1)	Disagree (2)	Neutral (3)	Agree (4)	Strongly agree (5)
I am excited about the Simodont	4.725	0.554	0 (0%)	0 (0%)	2 (5%)	7 (17.5%)	31 (77.5)
I expect Simodont to improve my clinical/preclinical skills	4.675	0.572	0 (0%)	0 (0%)	2 (5%)	9 (22.5%)	29 (72.5%)
I expect Simodont to be user friendly	3.80	0.822	0 (0%)	1 (2.5%)	15 (37.5%)	15 (37.5%)	9 (22.5%)
I expect to acquire manual dexterity tests quicker with Simodont than in the traditional preclinical training methods	3.825	0.957	0 (0%)	3 (7.5%)	13 (32.5%)	12 (30%)	12 (30%)
I expect to be able to integrate Simodont easily in my learning environment	3.85	0.802	0 (0%)	2 (5%)	10 (25%)	20 (50%)	8 (20%)
I expect added value in the use of Simodont in my dental training	4.65	0.533	0 (0%)	0 (0%)	1 (2.5%)	12 (30%)	27 (67.5%)
I expect working on Simodont is realistic	3.90	0.900	0 (0%)	2 (5%)	9 (22.5%)	17 (42.5%)	12 (30%)

**Table 2:** Showing means and standard deviations, and frequency distributions of answers of the students’ post-experimental questionnaire.

Question	Mean	SD	Strongly disagree (1)	Disagree (2)	Neutral (3)	Agree (4)	Strongly agree (5)
The images of anatomical models and instruments in Simodont looked realistic	3.825	1.03	2 (5%)	1 (2.5%)	10 (25%)	16 (40%)	11 (27.5%)
The hardness, texture and tactile feedback provided by Simodont felt realistic	4.65	0.57	0 (0%)	0 (0%)	2 (5%)	10 (25%)	28 (70%)
The instructions given by Simodont were clear and provided in an easy format	4.4	0.74	0 (0%)	1 (2.5%)	3 (7.5%)	15 (37.5%)	21 (52.5%)
I felt comfortable using Simodont	4.125	0.72	0 (0%)	1 (2.5%)	5 (12.5%)	22 (55%)	12 (30%)
Using Simodont assisted my learning	4.75	0.43	0 (0%)	0 (0%)	0 (0%)	10 (25%)	30 (75%)
The educational feedback provided by Simodont assisted my learning	4.625	0.58	0 (0%)	0 (0%)	2 (5%)	11 (27.5%)	27 (67.5%)
The force feedback provided by Simodont assisted my learning	4.625	0.62	0 (0%)	0 (0%)	3 (7.5%)	9 (22.5%)	28 (70%)
Using Simodont improved my Visual-motor skills	4.575	0.63	0 (0%)	0 (0%)	3 (7.5%)	11 (27.5%)	26 (65%)
Using Simodont improved my knowledge	4.15	0.94	0 (0%)	2 (5%)	9 (47.5%)	10 (25%)	19 (47.5%)
I feel more confident about my skills after using Simodont	4.175	0.84	0 (0%)	1 (2.5%)	8 (20%)	14 (35%)	17 (42.5%)
Using Simodont in labs in the future will help improve my preclinical/clinical skills	4.8	0.40	0 (0%)	0 (0%)	0 (0%)	8 (20%)	32 (80%)
Simodont should be offered to all dentistry students prior to performing procedures on real patients	4.875	0.33	0 (0%)	0 (0%)	0 (0%)	5 (12.5%)	35 (87.5%)
I would prefer feedback from Simodont to be supplemented by feedback from a tutor or a lecturer as well	4.625	0.70	0 (0%)	1 (2.5%)	2 (5%)	8 (20%)	29 (72.5%)
Simodont approximates a real preclinical experience to me	3.75	0.84	0 (0%)	3 (7.5%)	11 (27.5%)	19 (47.5%)	7 (17.5%)
Do you think Simodont would be a useful educational tool in Dental training programs?	4.725	0.50	0 (0%)	0 (0%)	1 (2.5%)	9 (22.5%)	30 (75%)
Simodont should totally replace Phantom heads in preclinical training	2.55	1.06	6 (15%)	15 (37.5%)	12 (30%)	5 (12.5%)	2 (5%)

**Figure 1:**
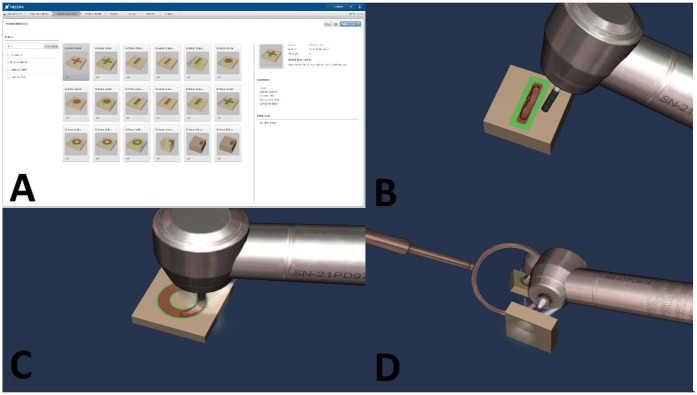
Showing examples of the manual dexterity exercises that were used in the evaluation sessions conducted in the present study. **(A)** The list of the variable manual dexterity exercises that are offered by the Simodont® Dental Trainer. **(B)** The linear manual dexterity exercise (direct vision). **(C)** The hollow circle (donut) manual dexterity exercise (direct vision). **(D)** The solid circle manual dexterity exercise (indirect vision). Images are courtesy of Nissin Dental Products Europe BV.

All tasks given to the participants during the evaluation session were identical and included manual dexterity exercises, clinical exercises on a single tooth as well as a simulated full arch experience with teeth present in contact. These tasks were also identical to the tasks completed by participants in the corresponding studies conducted over a decade ago within the same educational institution. Figure 1 demonstrates some examples of the manual dexterity exercises used in the evaluation sessions in the present study.

## Results

Cronbach's alpha was calculated to test reliability and internal consistency for ratings of the questions of the pre and post experimental questionnaires. Both questionnaires showed high reliability [alpha = 0.834 (pre), alpha = 0.925 (post)].

## Students' questionnaires

### Pre-experimental questionnaire

The results obtained from our pre-experimental questionnaire showed that the first-year students of the School of Medicine and Dentistry (Griffith University) were above neutral and almost positive in regard to their expectations of the Simodont® Dental Trainer. The element of excitement about exploring the virtual dental simulator rated the highest of all questions, followed by their expectations about the value of the Simodont® Dental Simulator in dental education and improvement of skills. The Mean values for the answers of all seven questions presented in the pre-experimental questionnaire ranged between 3.8 and 4.725.

### Post-experimental questionnaire

Following the trial of the Simodont® Dental Trainer, the first-year students remained positive about all aspects of the technology with a high degree of satisfaction showing in their responses with thirteen out of sixteen questions from the post-experimental questionnaire showing a mean response above 4. The realism of images and the approximation to a real traditional educational experience had a mean response below 4 (3.825 and 3.75 respectively). The consensus from their responses to the post-experimental questionnaire showed agreement about the educational benefits and value of the Simodont® Dental Trainer in pre-clinical training. The lowest rate out of all questions from the post-experimental questionnaire was related to the integration of this technology, where the first-year students disagreed to the Simodont® Dental Trainer totally replacing the traditional phantom heads in preclinical training. Table 2 summarizes the results obtained from the post experimental questionnaire.

## Academics' questionnaires

### Pre-experimental questionnaire

The results obtained from our pre-experimental questionnaire showed that the academic staff members of the School of Medicine and Dentistry – Griffith University overall positive about using the Simodont® Dental simulator. The Mean values for the answers to questions presented in the pre-experimental questionnaire ranged between 3.36 and 4.55. The expectations about the realism of the Simodont's virtual reality environment were rated the lowest by academic staff members.

Table 3 summarizes the results obtained from the academics' pre-experimental questionnaire.

**Table 3:** Showing means and standard deviations, and frequency distributions of answers of the academics’ pre-experimental questionnaire.

Question	Mean	SD	Strongly disagree (1)	Disagree (2)	Neutral (3)	Agree (4)	Strongly agree (5)
I am excited about the Simodont	4.55	0.69	0 (0%)	0 (0%)	1 (9.1%)	3 (27.3%)	7 (63.6%)
I expect Simodont to improve my students’ clinical/preclinical skills	4.27	0.65	0 (0%)	0 (0%)	1 (9.1%)	6 (54.5%)	4 (36.4%)
I expect Simodont to be user friendly	4.55	0.82	0 (0%)	0 (0%)	2 (18.2%)	2 (18.2%)	7 (63.6%)
I expect my students to acquire manual dexterity tests quicker with Simodont than in the traditional preclinical training methods	4.00	0.77	0 (0%)	0 (0%)	3 (27.3%)	3 (27.3%)	5 (45.4%)
I expect to be able to integrate Simodont easily in my students’ learning environment	4.55	0.69	0 (0%)	0 (0%)	1 (9.1%)	3 (27.3%)	7 (63.6%)
I expect added value in the use of Simodont in my students’ Dental training	4.55	0.52	0 (0%)	0 (0%)	0 (0%)	6 (54.5%)	5 (45.5%)
I expect working on Simodont is realistic	3.36	1.12	1 (9.1%)	1 (9.1%)	3 (27.3%)	5 (45.4%)	1 (9.1%)

### Post-experimental questionnaire

Comparing the statistical results obtained from the post-experimental questionnaire to the ones obtained from the pre-experimental showed that the Simodont® dental trainer was not up to the academic staff members' expectations in terms of the realism of the 3D models' appearance and texture as well as the educational benefits that virtual simulator offers in terms of knowledge acquisition. Despite the concerns noted above, the academic staff members seemed positive about continuing using the Simodont® dental trainer in their students' preclinical learning environment. A point of agreement between the students' and academic staff members' responses to the post-experimental questionnaire was the value of the human element in the educational process where all participants (students and staff) agreed that the feedback from the Simodont® dental trainer should be augmented with instructions from a human supervisor and that virtual dental simulation should be offered parallel to rather than as a replacement to traditional manikin based simulation.

Table 4 summarizes the results obtained from the academics' post experimental questionnaire.

**Table 4:** Showing means and standard deviations, and frequency distributions of answers of the academics’ post-experimental questionnaire.

Question	Mean	SD	Strongly disagree (1)	Disagree (2)	Neutral (3)	Agree (4)	Strongly agree (5)
The images of anatomical models and instruments in Simodont looked realistic	4.00	0.77	0 (0%)	0 (0%)	3 (27.3%)	5 (45.4%)	3 (27.3%)
The hardness, texture and tactile feedback provided by Simodont felt realistic	3.73	0.90	0 (0%)	0 (0%)	6 (54.5%)	2 (18.2%)	3 (27.3%)
The instructions given by Simodont were clear and provided in an easy format	3.55	1.21	0 (0%)	3 (27.3%)	2 (18.1%)	3 (27.3%)	3 (27.3%)
I felt comfortable using Simodont	4.64	0.50	0 (0%)	0 (0%)	0 (0%)	4 (36.4%)	7 (63.6%)
Using Simodont assisted my students’ learning	4.18	0.75	0 (0%)	0 (0%)	2 (18.2%)	5 (45.4%)	4 (36.4%)
The educational feedback provided by Simodont assisted my students’ learning	2.90	1.64	3 (27.3%)	2 (18.2%)	2 (18.2%)	1 (9.1%)	3 (27.2%)
The force feedback provided by Simodont assisted my students’ learning	4.00	0.89	0 (0%)	1 (9.1%)	1 (9.1%)	6 (54.5%)	3 (27.3%)
Using Simodont improved my students’ Visual-motor skills	4.36	0.67	0 (0%)	0 (0%)	1 (9.1%)	5 (45.4%)	5 (45.5%)
Using Simodont improved my students’ knowledge	2.64	1.50	4 (36.4%)	1 (9.1%)	2 (18.2%)	3 (27.2%)	1 (9.1%)
I think my students will feel more confident about their skills after using Simodont	4.00	0.89	0 (0%)	1 (9.1%)	1 (9.1%)	6 (54.5%)	3 (27.3%)
Using Simodont in labs in the future will help improve my students’ preclinical/clinical skills	4.64	0.81	0 (0%)	0 (0%)	2 (18.2%)	0 (0%)	9 (81.8%)
Simodont should be offered to all dentistry students prior to performing procedures on real patients	4.55	0.93	0 (0%)	1 (9.1%)	0 (0%)	2 (18.2%)	8 (72.7%)
I would prefer feedback from Simodont to be supplemented by feedback from a tutor or a lecturer as well	4.64	0.92	0 (0%)	1 (9.1%)	0 (0%)	1 (9.1%)	9 (81.8%)
Simodont approximates a real preclinical experience to me	3.45	1.12	0 (0%)	3 (27.3%)	1 (9.1%)	4 (36.4%)	2 (18.2%)
Do you think Simodont would be a useful educational tool in Dental training programs?	4.45	0.68	0 (0%)	0 (0%)	1 (9.1%)	4 (36.4%)	6 (54.5%)
Simodont should totally replace Phantom heads in preclinical training	1.90	1.57	7 (63.6%)	2 (18.2%)	0 (0%)	0 (0%)	2 (18.2%)

## Comparative analysis with the results from our previous studies

### Comparative analysis of the students' pre-experimental questionnaire

Comparative analysis of the students' responses to the pre-experimental questionnaire from the current study vs. the corresponding questionnaire from the study by Bakr et al., 2014 (9) showed the current students provided significantly higher mean responses to the questions related to excitement, skill improvement, speed of skills acquisition compared to traditional methods, and added value in dental training when compared to the students from the last decade in the study conducted by Bakr et al., 2014 (9). Table 5 summarizes the results of the comparative analysis for the students’ pre-experimental questionnaire between the present study vs. the study by Bakr et al., 2014 (9).

**Table 5:** Showing the summary of the comparative analysis for the students’ pre-experimental questionnaires of the present study vs. the study by Bakr et al., 2014 (9).

Question	*P* value	95% confidence interval	*t*	df	Standard error of difference
I am excited about the Simodont	0.0301	0.03012 to 0.57988	2.2090	78	0.138
I expect Simodont to improve my clinical/preclinical skills	<0.0001	0.56751 to 1.14249	5.9208	78	0.144
I expect Simodont to be user friendly	0.0740	−0.67176 to 0.03176	1.8111	78	0.177
I expect to acquire manual dexterity tests quicker with Simodont than in the traditional preclinical training methods	0.0167	0.09405 to 0.91595	2.4465	78	0.206
I expect to be able to integrate Simodont easily in my learning environment	0.7822	−0.40881 to 0.30881	0.2774	78	0.180
I expect added value in the use of Simodont in my dental training	0.0108	0.09037 to 0.66963	2.6120	78	0.145
I expect working on Simodont is realistic	0.2086	−0.14250 to 0.64250	1.2681	78	0.197

### Comparative analysis of the students' post-experimental questionnaire

The were significant differences between the post-experimental responses obtained from the students in the current study vs. the students in the study by Bakr et al., 2014 (9) in almost all aspects of the evaluation of the virtual dental simulator. On the other hand, the areas of agreement between both students' cohorts were related to their evaluation of the realism of images, clarity of instructions and feedback provided by the simulator, as well as the importance of supplementing the virtual dental simulation experience with feedback from a human supervisor. It should be noted that the students from the current study rated all aspects of their experience with the Simodont® dental trainer significantly higher than the student cohort from the study by Bakr et al., 2014 (9). Furthermore, they were more accepting to the idea of virtual dental simulation replacing phantom heads in preclinical training in comparison to their counterparts in the 2014 study. Table 6 summarizes the results of the comparative analysis for the students' post-experimental questionnaire between the present study vs. the study by Bakr et al., 2014 (9).

**Table 6:** Showing the summary of the comparative analysis for the students’ post-experimental questionnaires of the present study vs. the study by Bakr et al., 2014 (9).

Question	*P* value	95% confidence interval	*t*	df	Standard error of difference
The images of anatomical models and instruments in Simodont looked realistic	0.4961	−0.29624 to 0.60624	0.6839	78	0.227
The hardness, texture and tactile feedback provided by Simodont felt realistic	<0.0001	1.33668 to 1.96332	10.4841	78	0.157
The instructions given by Simodont were clear and provided in an easy format	0.0413	0.01533 to 0.74467	2.0746	78	0.183
I felt comfortable using Simodont	<0.0001	0.56620 to 1.28380	5.1325	78	0.180
Using Simodont assisted my learning	<0.0001	1.08829 to 1.71171	8.9417	78	0.157
The educational feedback provided by Simodont assisted my learning	<0.0001	0.89627 to 1.61373	6.9649	78	0.180
The force feedback provided by Simodont assisted my learning	<0.0001	1.12429 to 1.78571	8.7590	78	0.166
Using Simodont improved my Visual-motor skills	<0.0001	0.87679 to 1.63321	6.6061	78	0.190
Using Simodont improved my knowledge	<0.0001	0.78948 to 1.61052	5.8195	78	0.206
I feel more confident about my skills after using Simodont	<0.0001	0.78917 to 1.52083	6.2855	78	0.186
Using Simodont in labs in the future will help improve my preclinical/clinical skills	<0.0001	0.82126 to 1.37874	7.8566	78	0.140
Simodont should be offered to all dentistry students prior to performing procedures on real patients	<0.0001	0.87857 to 1.47143	7.8913	78	0.149
I would prefer feedback from Simodont to be supplemented by feedback from a tutor or a lecturer as well	0.2501	−0.14724 to 0.55724	1.1587	78	0.177
Simodont approximates a real preclinical experience to me	<0.0001	0.51702 to 1.24298	4.8265	78	0.182
Do you think Simodont would be a useful educational tool in Dental training programs?	<0.0001	0.48568 to 1.16432	4.8403	78	0.170
Simodont should totally replace Phantom heads in preclinical training	<0.0001	0.90351 to 1.65649	6.7685	78	0.189

### Comparative analysis of the academics' pre-experimental questionnaire

Comparative analysis of the students' responses to the pre-experimental questionnaire from the current study vs. the corresponding questionnaire from the study by Bakr et al., 2013 (8) showed the current academic staff members has significantly higher expectations regarding the Simodont® dental trainer when compared to the academics from the last decade in the study conducted by Bakr et al., 2013 (8). The only exceptions were the user friendliness and realism of the Simodont® dental trainer where responses from academics from both studies showed no statistical difference, despite current participants rating both aspects higher than their 2013 counterparts. Table 7 summarizes the results of the comparative analysis for the academics’ pre-experimental questionnaire between the present study vs. the study by Bakr et al., 2013 (8).

**Table 7:** Showing the summary of the comparative analysis for the academics’ pre-experimental questionnaires of the present study vs. the study by Bakr et al., 2013 (8).

Question	*P* value	95% confidence interval	*t*	df	Standard error of difference
I am excited about the Simodont	0.0381	−1.6097 to −0.0503	2.2204	20	0.374
I expect Simodont to improve my students’ clinical/preclinical skills	0.0388	−1.7683 to −0.0517	2.2115	20	0.411
I expect Simodont to be user friendly	0.0837	−1.5879 to 0.1079	1.8206	20	0.406
I expect my students to acquire manual dexterity tests quicker with Simodont than in the traditional preclinical training methods	0.0046	−2.2667 to −0.4733	3.1868	20	0.430
I expect to be able to integrate Simodont easily in my students’ learning environment	0.0137	−1.7897 to −0.2303	2.7020	20	0.374
I expect added value in the use of Simodont in my students’ dental training	0.0118	−1.9283 to −0.2717	2.7701	20	0.397
I expect working on Simodont is realistic	0.5947	−1.3116 to 0.7716	0.5407	20	0.499

### Comparative analysis of the academics' post-experimental questionnaire

There were significant differences between the responses to the post-experimental questionnaire by the academics in the current study in comparison to their counterparts in the 2013 study by Bakr et al. (8), in the areas of the realism of the hardness, texture, tactile feedback provided by the Simodont® dental trainer, feeling comfortable while using the simulator, its usefulness as an educational tool, its value in improving clinical/preclinical skills and the necessity to offer its technology to all students prior to performing clinical procedures on real patients. Furthermore, there was a trend towards higher ratings by the academics in the current study when compared to the previous study by Bakr et al., 2013 (8), despite not being statistically significant for other aspects of the post-experimental questionnaire. Table 8 summarizes the results of the comparative analysis for the academics' post-experimental questionnaire between the present study vs. the study by Bakr et al., 2013 (8).

**Table 8:** Showing the summary of the comparative analysis for the academics’ post-experimental questionnaires of the present study vs. the study by Bakr et al., 2013 (8).

Question	*P* value	95% confidence interval	*t*	df	Standard error of difference
The images of anatomical models and instruments in Simodont looked realistic	0.8294	−0.7700 to 0.9500	0.2183	20	0.412
The hardness, texture and tactile feedback provided by Simodont felt realistic	0.0303	−2.0841 to −0.1159	2.3316	20	0.472
The instructions given by Simodont were clear and provided in an easy format	0.4387	−0.57392 to 1.27392	0.7902	20	0.443
I felt comfortable using Simodont	0.0001	−1.88378 to −0.85622	5.5623	20	0.246
Using Simodont assisted my students’ learning	0.0876	−1.5778 to 0.1178	1.7962	20	0.406
The educational feedback provided by Simodont assisted my students’ learning	0.3212	−0.6723 to 1.9523	1.0173	20	0.629
The force feedback provided by Simodont assisted my students’ learning	0.1326	−1.4914 to 0.2114	1.5680	20	0.408
Using Simodont improved my students’ Visual-motor skills	0.0784	−1.5508 to 0.0908	1.8551	20	0.394
Using Simodont improved my students’ knowledge	0.1265	−0.2774 to 2.0774	1.5945	20	0.564
I think my students will feel more confident about their skills after using Simodont	0.1535	−1.5397 to 0.2597	1.4838	20	0.431
Using Simodont in labs in the future will help improve my students’ preclinical/clinical skills	0.0314	−1.9206 to −0.0994	2.3137	20	0.437
Simodont should be offered to all dentistry students prior to performing procedures on real patients	0.0038	−2.09571 to −0.46429	3.2732	20	0.391
I would prefer feedback from Simodont to be supplemented by feedback from a tutor or a lecturer as well	0.2925	−1.3475 to 0.4275	1.0811	20	0.425
Simodont approximates a real preclinical experience to me	0.0757	−1.73320 to 0.09320	1.8731	20	0.438
Do you think Simodont would be a useful educational tool in Dental training programs?	0.0341	−1.9172 to −0.0828	2.2744	20	0.440
Simodont should totally replace Phantom heads in preclinical training	0.4236	−1.59896 to 0.69896	0.8170	20	0.551

## Open-ended comments

The themes observed in the open-ended comments from participants focused on applications in dental education as well as technical and educational limitations. Furthermore, there were some comments related to hardware components of the software.

### Examples of the open-ended responses include

“A cost-effective tool that allows repetitive training in a risk-free environment.”

“The experience with the virtual simulation flowed seamlessly and I was extremely engaged to the extent that I did not feel the flow of time.”

“The hardware design forced me to sit in the correct ergonomic position which was a great addition to my training.”

“I like the flexibility and the ability to access the virtual dental simulators any time for extra training.”

“I feel that prolonged exposure to the 3D virtual reality environment could cause headaches, vertigo and eye strain to some users.”

“There was a bit difference between the cutting efficiency of the sides and tip of the bur.”

“The variability in consistency between different parts of the target material made it extremely difficult to create a smooth floor.”

“The software interface has the ability to include more educational material to supplement the practical component offered by the simulator.”

“The joystick that manipulates the orientation of the objects in the virtual environment needs to provide more flexibility in movement.”

“The ratio between the sizes of the dental mirror and the handpiece needs to be finetuned.”

“The technology is impressive. However, it does not equip the students with the appreciation of human anatomical variation, infection control measures, empathy and communication skills.”

“The hardware design around the horseshoe finger rest area was artificial and did not feel natural to an experienced clinician who is used to intra-oral and extra-oral support while holding dental instruments.”

## Discussion

Over the past decade, dental education has undergone a significant transformation with the integration of virtual simulation technologies. What once began as a supplementary tool met with scepticisms and uncertainty has gradually evolved into a core component of preclinical training in many institutions (2, 14). In its early stages, virtual dental simulation faced challenges related to realism, accessibility, and user trust. However, advances in haptic feedback, immersive interfaces, and the growing demand for remote learning, especially during the COVID-19 pandemic, have shifted user perceptions dramatically (15). Today, these simulations are not only more widely accepted but are also seen as essential in bridging the gap between theoretical knowledge and clinical practice (16–18). This study explores the evolution of user perceptions and acceptance of virtual dental simulation, comparing attitudes and adoption rates from a decade ago to the present day, and examining the factors that have influenced this paradigm shift, including the technological progress, pedagogical integration, and broader cultural shifts in education as well as evaluates how these changes have impacted its effectiveness and legitimacy within dental training by comparing historical and contemporary viewpoints.

Since our previous studies over a decade ago, significant technological advancement, particularly in haptic feedback, graphical fidelity, and user interface design transformed virtual simulation into a far more immersive and credible training environment. Concurrently, the educational landscape itself has evolved, shaped in part by the global COVID-19 pandemic, which accelerated the demand for remote and flexible learning solutions. These shifts have prompted a re-evaluation of virtual simulation's role in dental education, with more recent cohorts of students and faculty demonstrating greater acceptance and even preference for these tools in preclinical learning.

Examining how user perceptions and acceptance of virtual simulation in dental education have evolved over a ten-year period offers critical insights into the intersection of technology, pedagogy, and user experience. A longitudinal perspective (19) allows researchers and educators to assess not only how technological tools have developed, but also how attitudes toward their use have shifted in response to changes in digital literacy, instructional needs, and institutional readiness (20). The comparative analysis over a decade is important in revealing whether technological advancements, such as improved haptic feedback, higher graphical fidelity, and enhanced interactivity have translated into greater perceived usefulness and trust among users. Consequently, if perceptions remain largely unchanged despite technological improvements, it may suggest that deeper issues including cultural, pedagogical, or logistical barriers are limiting widespread acceptance. One of the aspects that our present study highlights is the influence of generational change within both the student body and faculty (21, 22). As newer cohorts of students, often characterized as digital natives (23), enter dental education with different learning preferences and expectations, their acceptance of virtual tools may differ markedly from that of previous generations. Simultaneously, faculty attitudes toward educational technology may also evolve, influenced by professional development, institutional policy, and exposure to digital teaching tools.

Furthermore, a decade-long comparison captures the impact of broader contextual shifts in education, particularly those accelerated by events like the COVID-19 pandemic which forced many institutions to rapidly adopt virtual learning tools, including simulation, offering a unique opportunity to evaluate how necessity-driven exposure may have reshaped users' views on simulation-based training (24, 25). To our knowledge, this is the first longitudinal study of its kind in the field of virtual dental simulation. Moreover, such a temporal comparison enables a more nuanced evaluation of integration success. Rather than relying on isolated snapshots of opinion, a decade-long lens reveals whether simulation has become more deeply embedded in dental curricula, and whether users have moved from viewing it as a novelty to recognizing it as an essential component of clinical training. Finally, this type of research contributes to the broader field of educational technology adoption and diffusion by identifying long-term trends, challenges, and enabling factors. It informs policy makers, curriculum designers, and software developers on how to tailor future innovations to meet user expectations and pedagogical demands more effectively.

When originally introduced as a novel supplement to conventional hands-on training, early implementations of virtual dental simulation were met with cautious interest, often perceived as lacking the realism and tactile fidelity necessary for effective clinical skill development. Limited by early-stage technology and minimal integration into curricula, the initial reception among students and educators alike was mixed following classical patterns of adoption models in health education (26). During its formative years, virtual simulation was often perceived as an experimental supplement rather than a reliable substitute for traditional hands-on training. A number of early studies (8, 9, 27) reported concerns regarding technical limitations, high implementation costs, and insufficient tactile realism, which hindered widespread acceptance among both students and faculty.

The results from our study are consistent with technology adoption models including Theoretical Frameworks of Technology Acceptance including the Technology Acceptance Model (TAM) (28) which has been proven useful in predicting users' acceptance of certain technological aspects in health care (29), health education (30) and dentistry (31, 32), as well as the Unified Theory of Acceptance and Use of Technology (UTAUT) (33). Virtual dental simulation is now considered to have achieved the stage of mainstream integration and is currently undergoing the stages of maturity and standardization, increased user competency as well as refined use and optimization. Future stages will include data on long-term impact which is an area that is currently lacking in the literature, hence, the importance of the current study in providing data that covers a decade of virtual dental simulation with the use of the Simodont® dental trainer. Future stages of the adoption of virtual dental simulation will include evolution through major updates or integrations with newer innovations including artificial intelligence feedback and automation.

Understanding the evolution of user perceptions toward virtual dental simulation necessitates an examination of the generational shifts that have occurred within dental education over the past decade. The differing levels of familiarity, comfort, and expectations regarding technology between past and current learners are critical to explaining the variations in acceptance of virtual simulation learning environments. Prensky's (2001) (34) Digital Native Theory provides a useful framework for conceptualizing these generational differences. Digital natives, typically individuals born after 1980, have been immersed in technology from a young age and thus exhibit a high degree of fluency with digital tools. In contrast, earlier cohorts of dental students and faculty, often categorized as digital immigrants, had to adapt to technological innovations later in life (23, 34). As such, early perceptions of virtual dental simulation were often marked by scepticism and resistance, particularly due to perceived deficiencies in realism, tactile feedback, and the unfamiliar nature of the interface (35). It should be noted that despite the potential perceived bias due to different sample characteristics at different times, there were lots of similarities between the results obtained from the current study when compared to our previous studies that were conducted a decade ago.

Contemporary dental students, as digital natives, generally demonstrate greater openness to technology-enhanced learning. Studies in healthcare education have highlighted that current learners often prefer interactive, self-paced, and experiential learning modalities (36–39), which align closely with the capabilities of virtual simulation platforms. This shift in learning preferences supports higher acceptance rates and greater engagement with simulation technologies among recent student cohorts compared to those of a decade ago. Moreover, expectations surrounding educational technology have changed. Virtual tools are no longer perceived as supplemental or experimental; rather, they are increasingly viewed as integral to the delivery of high-quality education (40–42). The widespread call for adoption of online and remote learning platforms during the COVID-19 pandemic further normalized the use of virtual technologies in clinical education (43). We believe this potentially reinforces a more positive perception of their value and legitimacy among digital-native students. Despite these generational shifts, certain critiques of virtual dental simulation persist (5). Both earlier and current users have expressed concerns regarding the lack of tactile realism and the limited scope of patient variability offered by simulation platforms (5, 8, 9). These enduring limitations suggest that while generational factors contribute to greater acceptance, technological shortcomings remain a barrier to full integration, regardless of a user's digital literacy.

An additional layer of complexity arises when considering faculty attitudes. Generational differences between students and instructors can influence the institutional climate surrounding virtual simulation. Faculty members from earlier generations may exhibit lower levels of enthusiasm or confidence in digital teaching tools, which can indirectly shape student perceptions (44). Resistance among some faculty members will always persist as a barrier to full acceptance. Educators who are less familiar with or confident in using simulation technologies may be hesitant to integrate them fully into the curriculum, thereby reinforcing conservative views among students and slowing broader cultural acceptance. Faculty hesitance, rooted in both technological discomfort and pedagogical tradition, has remained a notable constant in the discourse surrounding simulation in dental education. Conversely, younger or more technologically adept educators may champion the integration of simulation technologies, fostering a more supportive learning environment. The current study followed the same protocol as our previous studies a decade ago for a sound comparative analysis, and did not take into account the age or graduation year of the academic staff members as the main focus of our study was the change in the acceptance or perception of the Simodont® Dental Trainer over a decade. We recommend that future studies explore the impact of generational differences on the adoption of virtual simulation in dental education.

Despite increasing familiarity and integration within dental curricula, recurring concerns, particularly around the authenticity of the simulation experience continue to shape user attitudes. A primary reason for the persistent scepticism lies in the limitations of tactile realism and haptic feedback. Many users, both historically and presently, have expressed concern that current simulation platforms do not accurately replicate the nuanced tactile sensations of working within the oral cavity (8, 9, 45). Despite improvements in hardware and interface design, the inability of virtual systems to fully emulate tissue resistance, anatomical variability, and fine motor responses continues to hinder their perceived equivalency to traditional hands-on training. This limitation is particularly salient in a discipline like dentistry, where psychomotor precision is critical and clinical confidence is often built through physical repetition. Furthermore, infection control, ethics and communication skills, being integral domains in the dental profession, are not assessed nor developed using virtual dental simulation which remains a drawback in the technology (5), that was reflected in the open-ended responses the participants provided in the present study. However, some work in developing in the health education landscape to include ethical dilemmas in the world of virtual simulation (46). Moreover, the lack of real patient interaction in virtual environments continues to be a point of critique. While virtual simulations provide structured, standardized cases, they often fail to capture the unpredictability, interpersonal dynamics, and contextual variability present in live clinical settings. We hypothesize that the perceived detachment from the real-world patient experience contributes to ongoing reservations about the ability of simulation alone to prepare students adequately for clinical encounters.

Another factor that has remained relatively consistent is the perceived cost-benefit ratio of simulation tools (5). Even with broader institutional support and decreasing hardware costs, the initial investment required for simulation technology along with the associated faculty training, maintenance, and software updates remains a concern (5), particularly in resource-constrained educational environments. For institutions and educators, these considerations continue to affect the perceived feasibility and scalability of virtual simulation in dental education. Furthermore, the lack of consistency between virtual dental simulation tools as well as the studies evaluating their performance and efficacy (47), remains a concern for educational institutions when making a decision to invest in these technologies due to the absence of high-quality evidence based long term studies or a universal assessment tools/standards to validate new emerging virtual dental simulation technologies (5).

Additionally, assessment validity and feedback quality remain areas where user perceptions have changed little. While virtual systems often include automated performance metrics and scoring algorithms, users frequently question the depth, nuance, and clinical relevance of such feedback compared to that provided by experienced instructors. This scepticism may contribute to a lingering sense that simulation, while useful for skill acquisition and repetition, cannot fully replace expert-guided assessment and mentorship, which was consistent with our results in the present study as well as our previous studies a decade ago.

The advent of training with haptic devices may become a necessary part of training for dental graduates. These techniques can translate directly into apparatus that utilise robotics for a variety of dental procedures. Recent advances have seen implant dentistry incorporate haptic feed-back robotics for implant placement (48–50). Applications for tooth preparation, prosthetics, and crown lengthening procedures have also been noted (51, 52). These emerging technologies and techniques may cement the use of virtual simulation training as a necessity for training of current and future dental graduates. The inclusion of this modality of training may become even more integral to dental curriculum with the advent of robotic guided dental procedures, which may rely on a degree of user control via haptic feedback. Current shift in clinical practice that have seen robotics or automated systems to perform clinical procedures will also make this training current. The need for future dental graduates to be able to operate and oversee these systems is likely a critical future skill.

In addition to robotics technology, there could be some possible links between the findings from the current study and mixed reality technologies. Mixed reality (MR) is emerging as a transformative tool in dental education, blending the physical and digital worlds to enhance learning experiences. By integrating virtual elements with real-world interactions (53). MR allows students to visualize complex anatomical structures in 3D, simulate procedures with lifelike precision, and interact with digital overlays while working on physical models or patients (53). This fusion of virtual and real environments promotes deeper understanding, improves spatial awareness, and supports more effective clinical decision-making (54). As a result, mixed reality not only enriches traditional teaching methods but also bridges the gap between theory and hands-on practice in dental training (53–55).

The responses to the open-ended questions in the present study highlighted the value of virtual dental simulation in providing repetitive training in a safe environment which would be helpful to restore the self-confidence for students who are struggling with hand skills in tailor-made academic recovery programs (5). The ease of accessibility to virtual dental simulation with immediate feedback was also highlighted and appreciated by participants as one of the common themes. On the downside, despite the developments of the Simodont® dental trainer' software, the trend of receiving comments related to technical issues remained evident in the current study as seen in the previous study conducted over a decade ago (9). This could be contributed to the continuous need for further developments in the simulator's software as well as the possibility that users' expectations could have increased recently with lots of technological advances becoming more mainstream when compared to the last decade. Furthermore, while the value of the simulator's ability to develop practical hand skills was evident, the software's interface still requires more advancement in providing an additional value as an educational tool to improve the students' didactic knowledge. Finally, despite its ability to develop good habits in early learners when it comes to ergonomic positions, the Simodont® dental trainer lacked the ability to teach students about human anatomical variation, infection control measures, empathy and communication skills as identified by participants' responses in the open-ended questions in the current study. This highlights the fact that participants from both studies compared were in total agreement that the Simodont® dental trainer would not replace traditional preclinical/clinical teaching methods and should include the human involvement of supervisors to complement those missing elements in the educational process.

The present study highlights the importance of virtual dental simulation as a risk-free immersive tool in dental education. This technology enhances learning outcomes, builds confidence, and bridges the gap between theoretical knowledge and practical application, ultimately leading to better-prepared dental professionals. Our study fills a gap in the knowledge related to the lack of long-term comparative studies in the field of applications of virtual simulation in dental education. However, more gaps in the literature remain unexplored including the limited cross-cultural or multi-institutional research and minimal research on user perceptions beyond students, including administrators and regulating/accreditation bodies.

## Conclusions

Generational differences shaped by digital fluency, evolving pedagogical preferences, and shifting cultural expectations play a substantial role in influencing how users perceive and accept virtual dental simulation. While the current generation of learners exhibits greater receptivity, persistent concerns regarding realism, patient interaction, cost-effectiveness, feedback and instructional quality, have persisted over the past decade. These concerns indicate that perceptions are also shaped by the intrinsic capabilities and limitations of the technology itself. These enduring perceptions underscore the importance of addressing not only technological development but also pedagogical integration, faculty training, and curricular alignment to fully realize the potential of simulation-based dental education.

## Data Availability

The original contributions presented in the study are included in the article/Supplementary Material, further inquiries can be directed to the corresponding author.

## References

[1] Roy E Bakr MM George R. The need for virtual reality simulators in dental education: a review. Saudi Dent J. (2017) 29(2):41–7. 10.1016/j.sdentj.2017.02.0012 28490842 PMC5411891

[2] Li Y Ye H Ye F Liu Y Lv L Zhang P The current situation and future prospects of simulators in dental education. J Med Internet Res. (2021) 23(4):e23635. 10.2196/23635 33830059 PMC8063092

[3] Felszeghy S Liukkonen M Flacco N Bakr MM Rampf S Schick S-G Establishing the VR-haptic thinkers group: insights and progress in dental training technologies. Saudi Dental J. (2024) 36(12):1655–9. 10.1016/j.sdentj.2024.11.008 PMC11976090 40952862

[4] Moussa R Alghazaly A Althagafi N Eshky R Borzangy S. Effectiveness of virtual reality and interactive simulators on dental education outcomes. Systematic Review. Eur J Dent. (2022) 16(1):14–31. 10.1055/s-0041-1731837 34428851 PMC8890935

[5] Bakr MM Idris G Al Ankily M. The potential integration of Simodont® Dental Trainer in different stages of the dental curriculum. Saudi Dent J. (2024) 36(11):1449–55. 10.1016/j.sdentj.2024.09.002 39619714 PMC11605717

[6] Karanth D Arqub SA Dolce C. The applications of digital technology in postgraduate orthodontic education. Semin Orthod. (2024) 30(4):436–42. 10.1053/j.sodo.2024.03.003

[7] Khalid T Yaqoob H Syed FA Kazmi SMR. Assessing availability and trainees’ perceptions of simulation and augmented reality in prosthodontics postgraduate education in Pakistan: a cross-sectional study. BMC Med Educ. (2024) 24(1):1541. 10.1186/s12909-024-06542-4 39731123 PMC11681706

[8] Bakr MM Massey WL Alexander H. Evaluation of Simodont® haptic 3D virtual reality dental training simulator. Int J Dent Clin. (2013) 5:1–6.

[9] Bakr MM Massey WL Alexander H. Students’ evaluation of a 3DVR haptic device (Simodont®). does early exposure to haptic feedback during preclinical dental education enhance the development of psychomotor skills? Int J Dent Clin. (2014) 6(2014):1–7.

[10] Bakr MM Massey WL Alexander H. Can virtual simulators replace traditional preclinical teaching methods: a students’ perspective? Int J Dent Oral Health. (2015) 2(1):1–6. 10.16966/2378-7090.149

[11] Wei Y Peng Z. Application of Simodont virtual simulation system for preclinical teaching of access and coronal cavity preparation. PLoS One. (2024) 19(12):e0315732. 10.1371/journal.pone.0315732 39671406 PMC11642952

[12] Plessas A. Computerized virtual reality simulation in preclinical dentistry: can a computerized simulator replace the conventional phantom heads and human instruction? Simul Healthc. (2017) 12(5):332–8. 10.1097/SIH.0000000000000250 28697057

[13] Mirghani I Mushtaq F Allsop MJ Al-Saud LM Tickhill N Potter C Capturing differences in dental training using a virtual reality simulator. Eur J Dent Educ. (2018) 22(1):67–71. 10.1111/eje.12245 27864856

[14] Daud A Matoug-Elwerfelli M Khalid A Ali K. The impact of virtual reality haptic simulators in pre-clinical restorative dentistry: a qualitative enquiry into dental students’ perceptions. BMC Oral Health. (2024) 24(1):988. (Published August 23, 2024). 10.1186/s12903-024-04704-w 39180025 PMC11344466

[15] Fu Y Chu F Lu X Wang C Xiao N Jiang J Assessment and evaluation of online education and virtual simulation technology in dental education: a cross-sectional survey. BMC Med Educ. (2024) 24(191). 10.1186/s12909-024-05171-1 PMC10895829 38403582

[16] Sim JJM Rusli KDB Seah B Levett-Jones T Lau Y Liaw SY. Virtual simulation to enhance clinical reasoning in nursing: a systematic review and meta-analysis. Clin Simul Nurs. (2022) 69:26–39. 10.1016/j.ecns.2022.05.006 35754937 PMC9212904

[17] Medel D Reguant M Cemeli T Jiménez Herrera M Campoy C Bonet A Analysis of knowledge and satisfaction in virtual clinical simulation among nursing students: a mixed study. Nurs Rep. (2024) 14(2):1067–78. (Published April 27, 2024). 10.3390/nursrep14020081 38804414 PMC11130862

[18] Medel D Bonet A Herrera MJ Sevilla F Vilaplana J Cemeli T Interactive virtual simulation case: a learning environment for the development of decision-making in nursing students. Teach Learn Nurs. (2025) 20(1):e60–e8. 10.1016/j.teln.2024.08.002

[19] Caruana EJ Roman M Hernández-Sánchez J Solli P. Longitudinal studies. J Thorac Dis. (2015) 7(11):E537–40. 10.3978/j.issn.2072-1439.2015.10.63 26716051 PMC4669300

[20] Van Belle G Fisher LD Heagerty PJ Lumley T. Biostatistics: A Methodology for the Health Sciences. Hoboken, NJ: John Wiley & Sons (2004).

[21] Twenge JM. Generational changes and their impact in the classroom: teaching generation me. Med Educ. (2009) 43(5):398–405. 10.1111/j.1365-2923.2009.03310.x 19422486

[22] Lindskog H Oskarson MJWEP. Generational differences in disguise? A longitudinal study of the liberalising effect of education on socio-cultural attitudes. West Eur Polit. (2023) 46(3):500–25. 10.1080/01402382.2022.2076963

[23] Mertala P López-Pernas S Vartiainen H Saqr M MJCiHB T. Digital natives in the scientific literature: a topic modeling approach. Comput Human Behav. (2024) 152:108076. 10.1016/j.chb.2023.108076

[24] Aldekhyl SS Arabi YM. Simulation role in preparing for COVID-19. Ann Thorac Med. (2020) 15(3):134–7. 10.4103/atm.ATM_114_20 32831934 PMC7423200

[25] Dhaussy J Kemken L Pugliese M-T Forestier A Boloré SJT. Using simulation to adapt nursing education to times of crisis: a scoping review during COVID-19 pandemic. Teach Learn Nurs. (2024) 19(3):e511-7. 10.1016/j.teln.2024.03.003

[26] Pusic MV Ellaway RH. Researching models of innovation and adoption in health professions education. Med Educ. (2024) 58(1):164–70. 10.1111/medu.15161 37495269

[27] Smith SJ Farra SL Ulrich DL Hodgson E Nicely S Mickle A. Effectiveness of two varying levels of virtual reality simulation. Nurs Educ Perspect. (2018) 39(6):E10–5. 10.1097/01.NEP.0000000000000369 30335708

[28] Holden RJ Karsh BT. The technology acceptance model: its past and its future in health care. J Biomed Inform. (2010) 43(1):159–72. 10.1016/j.jbi.2009.07.002 19615467 PMC2814963

[29] Rahimi B Nadri H Lotfnezhad Afshar H Timpka T. A systematic review of the technology acceptance model in health informatics. Appl Clin Inform. (2018) 9(3):604–34. 10.1055/s-0038-1668091 30112741 PMC6094026

[30] Liu F Chang X Zhu Q Huang Y Li Y Wang H. Assessing clinical medicine students’ acceptance of large language model: based on technology acceptance model. BMC Med Educ. (2024) 24(1):1251. 10.1186/s12909-024-06232-1 39490999 PMC11533422

[31] Bahanan L Alsharif M. Factors affecting the acceptance of teledentistry determined using the technology acceptance model: a cross-sectional study. Digit Health. (2023) 9:20552076231158034. 10.1177/20552076231158034 36825078 PMC9941601

[32] Talla PK Kamalabadi YM Durand R Michaud PL Emami E. Applying an extended theoretical approach to identifying Canadian dental students’ acceptance of teledentistry: a cross-sectional study. Digit Health. (2024) 10:20552076241258472. 10.1177/20552076241258472 39351315 PMC11440558

[33] Xue L Rashid AM Ouyang S. The unified theory of acceptance and use of technology (UTAUT) in higher education: a systematic review. Saje Open. (2024) 14(1):21582440241229570. 10.1177/21582440241229570

[34] Prensky M. Digital natives, digital immigrants part 1. On the Horizon. (2001) 9(5):1–6. 10.1108/10748120110424816

[35] Gal GB Weiss EI Gafni N Ziv A. Preliminary assessment of faculty and student perception of a haptic virtual reality simulator for training dental manual dexterity. J Dent Educ. (2011) 75(4):496–504. 10.1002/j.0022-0337.2011.75.4.tb05073.x 21460270

[36] Evans KH Thompson AC O'Brien C Bryant M Basaviah P Prober C An innovative blended preclinical curriculum in clinical epidemiology and biostatistics: impact on student satisfaction and performance. Acad Med. (2016) 91(5):696–700. 10.1097/ACM.0000000000001085 26796089

[37] Ali NS John B. Examining the efficacy of online self-paced interactive video-recordings in nursing skill competency learning: seeking preliminary evidence through an action research. Med Sci Educ. (2019) 29(2):463–73. 10.1007/s40670-019-00714-4 34457503 PMC8368744

[38] Mukurunge E Reid M Fichardt A Nel M. Interactive workshops as a learning and teaching method for primary healthcare nurses. Health SA. (2021) 26:1643. (Published December 10, 2021). 10.4102/hsag.v26i0.1643 34956654 PMC8678960

[39] Oofuvong M Prathep S Plansangkate P Tanasansuttiporn J Sungworawongpana C Jitpakdee W. Self-study and online interactive case-based discussion to improve knowledge of medical students in the COVID-19 era. BMC Med Educ. (2024) 24(1):576. 10.1186/s12909-024-05578-w 38796438 PMC11128110

[40] Algarni YA Saini RS Vaddamanu SK Quadri SA Gurumurthy V Vyas R The impact of virtual reality simulation on dental education: a systematic review of learning outcomes and student engagement. J Dent Educ. (2024) 88(11):1549–62. 10.1002/jdd.13619 38807268

[41] Bandiaky ON Lopez S Hamon L Clouet R Soueidan A Le Guehennec L. Impact of haptic simulators in preclinical dental education: a systematic review. J Dent Educ. (2024) 88(3):366–79. 10.1002/jdd.13426 38044266

[42] Hadjichristou C Kokoti M Bakopoulou A. Haptics in fixed prosthodontics and their role in dental education: a literature review. J Dent Educ. (2024) 88(8):1020–8. 10.1002/jdd.13533 38558060

[43] Donovan SK Herstein JJ Prober CG Kolars JC Gordon JA Boyers P Expansion of simulation and extended reality for undergraduate health professions education: a call to action. J Interprof Educ Pract. (2021) 24:100436. 10.1016/j.xjep.2021.100436 36567809 PMC9765302

[44] Marrero Galván JJ Negrín Medina M Bernárdez-Gómez A Portela Pruaño A. The impact of the first millennial teachers on education: views held by different generations of teachers. Educ Inf Technol. (2023) 19:1–22. 10.1007/s10639-023-11768-8 PMC10113736 37361824

[45] Al-Saud LM Mushtaq F Allsop MJ Culmer PC Mirghani I Yates E Feedback and motor skill acquisition using a haptic dental simulator. Eur J Dent Educ. (2017) 21(4):240–7. 10.1111/eje.12214 27324833

[46] Rasesemola RM Molabe MPT. Enhancing student nurses’ ethical skills via simulation-based learning: barriers and opportunities. BMC Nurs. (2025) 24(1):147. 10.1186/s12912-025-02742-5 39920632 PMC11806624

[47] Baxmann M Baráth Z Kárpáti K. Efficacy of typodont and simulation training in orthodontic education: a systematic review. BMC Med Educ. (2024) 24(1):1443. 10.1186/s12909-024-06425-8 39695555 PMC11653705

[48] Neugarten JM. The use of robotics in implant dentistry. Oral Maxillofac Surg Clin. (2025) 37(2):341–52. 10.1016/j.coms.2024.11.004 39730288

[49] Alqutaibi AY Hamadallah HH Aloufi AM Tarawah RA. Applications of robots in implant dentistry: a scoping review. J Prosthet Dent. (2023) (In Press). 10.1016/j.prosdent.2023.11.019 38087758

[50] Bolding SL Reebye UN. Accuracy of haptic robotic guidance of dental implant surgery for completely edentulous arches. J Prosthet Dent. (2022) 128(4):639–47. 10.1016/j.prosdent.2020.12.048 33678441

[51] Alqutaibi AY Hamadallah HH Alturki KN Aljuhani FM Aloufi AM Alghauli MA. Practical applications of robots in prosthodontics for tooth preparation and denture tooth arrangement: a scoping review. J Prosthet Dent. (2025) 134(2):377.e1–e9. 10.1016/j.prosdent.2024.02.006 38480014

[52] Li Y Lyu J Cao X Zheng M Zhou Y Tan J Liu X. Development and accuracy assessment of a crown lengthening surgery robot for use in the esthetic zone: an *in vitro* study. J Prosthet Dent. (2024) (In Press). 10.1016/j.prosdent.2024.07.037 39155169

[53] Stevanie C Ariestiana YY Hendra FN Anshar M Boffano P Forouzanfar T Advanced outcomes of mixed reality usage in orthognathic surgery: a systematic review. Maxillofac Plast Reconstr Surg. (2024) 46(1):29. 10.1186/s40902-024-00440-x 39073682 PMC11286605

[54] Pelok SD Jasinevicius TR Turkyilmaz I. Taking preclinical dental education to another level with mixed reality technology. J Dent Sci. (2025) 20(2):1333–4. 10.1016/j.jds.2025.01.011 40224105 PMC11993095

[55] Blanchard J Koshal S Morley S McGurk M. The use of mixed reality in dentistry. Br Dent J. (2022) 233(4):261–5. 10.1038/s41415-022-4451-z 36028682

